# An Agentic Multimodal Sensing Architecture for CT-Guided Wearable and Respiratory Monitoring in Oncology Care

**DOI:** 10.3390/s26154817

**Published:** 2026-07-29

**Authors:** Denisa-Daniela Frimu-Pascu, Ciprian Dobre, Mihai Olteanu

**Affiliations:** 1 Faculty of Automatic Control and Computer Science, National University of Science and Technology Politehnica Bucharest, 060042 Bucharest, Romania; 2 Romanian Academy of Sciences, 030167 Bucharest, Romania; 3 National Institute for Research and Development in Informatics, 011455 Bucharest, Romania; 4 Discipline of Internal Medicine-Pneumology, University of Medicine and Pharmacy of Craiova, 200349 Craiova, Romania; mihai.olteanu@umfcv.ro

**Keywords:** intelligent sensing, AI-powered data processing, multimodal sensor fusion, agentic AI, oncology monitoring, pneumology, wearable sensors, CT contours, explainable AI, clinical decision support

## Abstract

Oncology care increasingly depends on heterogeneous sensing streams generated by computed tomography (CT), radiotherapy planning systems, wearable devices, home respiratory sensors, patient-reported outcomes, and clinical records. These data streams are often processed separately, limiting their value for longitudinal, context-aware review. This study proposes OncoSense-Agent, a reliability-aware agentic multimodal sensing architecture for CT-guided respiratory monitoring in oncology care. The architecture links CT-derived anatomical evidence with wearable physiology, respiratory symptoms, functional assessment, treatment context, and explainable human-in-the-loop review-priority generation. To move beyond a purely conceptual design, we implemented a lung-focused proof-of-concept with six bounded software agents: Imaging Reliability, Wearable Monitoring, Respiratory Review, Treatment Context, Multimodal Fusion, and Explainability. The prototype used real nnU-Net v2 3D lung segmentation metrics from 139 patients with complete bilateral lung CT data as the imaging anchor, while wearable, respiratory, symptom, and treatment-context channels were introduced as deterministic overlays for controlled validation. OncoSense-Agent changed review-priority assignment relative to CT-only assessment in 78/139 cases (56.1%), assigned 111/139 cases (79.9%) to high-priority or high-uncertainty tiers, and showed increasing Safety Gate activation as CT quality declined. Three illustrative cases demonstrate hidden respiratory deterioration, wearable data-quality uncertainty, and treatment-context risk not captured by CT-only assessment. The prototype does not establish clinical diagnostic accuracy, but demonstrates operational, auditable, reliability-aware multimodal review-priority generation for clinician-supervised oncology monitoring.

## 1. Introduction

Modern oncology care is increasingly shaped by multimodal sensing. Patients generate clinically relevant information through CT imaging, radiotherapy planning systems, electronic health records, wearable devices, home respiratory sensors, and patient-reported outcomes. In radiotherapy, CT defines tumors, organs at risk, body contours, and spatial relationships for treatment planning, while wearable and respiratory streams may capture functional change between clinical visits.

Despite this data richness, oncology workflows commonly process these streams separately. CT contours are used mainly during planning, wearable data are often reviewed in isolated dashboards, and symptoms are captured through separate questionnaires or interviews. This fragmentation can limit clinicians’ ability to relate anatomical risk, treatment exposure, physiological response, and patient experience over time.

This separation is particularly relevant in thoracic oncology, where CT is episodic and wearable or respiratory signals are longitudinal but noisy. A decline in oxygen saturation, activity, sleep, respiratory rate, dyspnea, cough, or functional capacity may reflect treatment toxicity, comorbidity, infection, progression, device artifact, or adherence issues. These signals therefore require reliability-aware interpretation rather than isolated thresholding.

OncoSense-Agent addresses this problem through bounded agents that coordinate imaging reliability, wearable monitoring, respiratory review, treatment context, multimodal fusion, and explainability. The goal is not autonomous diagnosis or treatment adaptation, but clinician-supervised review-priority generation with explicit uncertainty handling.

This study proposes OncoSense-Agent, an agentic multimodal sensing architecture that links CT-derived anatomical evidence, wearable physiology, respiratory monitoring, patient-reported symptoms, and treatment context within a human-in-the-loop workflow. A real thoracic CT segmentation evaluation is used as the imaging anchor, showing why automatic contours should be treated as reliability-scored sensing evidence rather than static ground truth.

The novelty is architectural and safety-oriented rather than algorithmic. OncoSense-Agent connects established components—CT segmentation, wearable monitoring, respiratory questionnaires, treatment-context reasoning, explainability, and governance—through reliability-aware orchestration and Safety Gate escalation.

The main contributions of this study are as follows:

1. A reliability-aware agentic multimodal sensing architecture for thoracic oncology and pneumology-oriented monitoring. OncoSense-Agent integrates CT-derived anatomical evidence, wearable physiology, respiratory measurements, patient-reported symptoms, treatment context, and clinician-facing explanations within a human-in-the-loop workflow.

2. An implemented six-agent proof-of-concept. The prototype includes bounded software agents for imaging reliability, wearable monitoring, respiratory review, treatment-context reasoning, multimodal fusion, and explainability, each with defined inputs, processing logic, outputs, reliability handling, and safety boundaries.

3. A real CT segmentation anchor for reliability-aware imaging interpretation. The proof-of-concept uses real nnU-Net v2 3D lung segmentation metrics from 139 patients with complete bilateral lung data, transforming CT contours from static outputs into reliability-scored anatomical sensing evidence.

4. Prototype-level validation of review-priority generation. The evaluation compares CT-only and multimodal review-priority assignments, reports Safety Gate behavior across CT quality tiers, and uses three illustrative cases to show how multimodal fusion can reveal hidden deterioration or uncertainty not captured by imaging alone.

The remainder of this study is organized as follows. Section 2 summarizes the background and gap-derived architecture requirements. Section 3 describes the proof-of-concept design, real CT data integration, deterministic multimodal overlays, implemented agents, fusion logic, and validation boundaries. Section 4 presents cohort-level and case-based validation results. Section 5 discusses the architectural and clinical implications. Section 6 presents limitations and future work. Section 7 concludes the study.

## 2. Background and Gap-Derived Architecture Requirements

### 2.1. CT Contours as Anatomical Sensing Outputs

CT imaging can be understood as a high-dimensional clinical sensing modality. In radiotherapy, CT defines tumors, organs at risk, body contours, and spatial relationships used for dose-volume analysis, quality assurance, and safety evaluation. Because constraints depend on correct anatomical boundaries, contour reliability is clinically important [1,2].

Deep learning has improved automatic medical image segmentation. U-Net, nnU-Net, Swin UNETR, TotalSegmentator, MedSAM, MONAI, V-Net, UNet++, DiNTS, generalized Dice loss, early stopping, and Adam optimization illustrate the maturity of this ecosystem [3,4,5,6,7,8,9,10,11,12,13,14]. However, performance remains organ-dependent, and segmentation outputs still require reliability estimation before downstream clinical use.

### 2.2. Wearable Sensors as Longitudinal Patient-State Signals

Wearable devices and smartwatches can collect physiological and behavioral signals outside the hospital, including heart rate, heart-rate variability, activity, sleep, oxygen saturation, respiratory rate, temperature, and electrocardiographic alerts when available. In oncology, such signals are increasingly studied for treatment monitoring, symptom tracking, rehabilitation planning, and remote patient monitoring [15,16,17,18,19].

Their value lies in capturing changes that occur between visits, but these data are noisy, incomplete, device-dependent, and context-sensitive. Oncology and remote-monitoring studies emphasize feasibility, adherence, data quality, and workflow integration as recurring implementation barriers [16,17,18,19,20,21]. Wearable signals should therefore be interpreted relative to baseline, treatment phase, symptoms, anatomical risk, and clinical context.

### 2.3. Pneumology-Oriented Respiratory Monitoring

Pneumology adds an important clinical perspective because thoracic cancer patients may have pre-existing or treatment-related respiratory vulnerability. Remote respiratory monitoring may include oxygen saturation, respiratory rate, symptoms, functional measures, physical activity, and spirometry; machine-learning evidence in COPD monitoring remains heterogeneous and requires careful validation [22,23].

In the proposed respiratory follow-up scenario, OncoSense-Agent can combine CT-derived lung contours, radiotherapy dose context, wearable oxygen saturation, respiratory rate, heart-rate trends, activity, sleep, dyspnea, cough, optional spirometry or peak-flow measures, 6MWT [24], mMRC [25], and CAT [26]. The objective is not autonomous diagnosis of pneumonitis, COPD exacerbation, infection, embolism, or progression, but prioritization of pneumology review when multimodal evidence deviates from baseline [27].

### 2.4. Multimodal Biomedical AI and Digital Patient-State Modeling

Multimodal biomedical AI integrates complementary data sources such as imaging, signals, text, structured clinical variables, and patient-generated health data [28]. In oncology, no single modality fully represents patient state: imaging provides anatomy, treatment plans provide exposure, wearables provide longitudinal physiology, symptoms provide patient experience, and clinical records provide context.

Digital patient-state modeling offers a practical way to organize these signals. In this work, the patient-state model is not a full biological simulation, but a structured representation of anatomy, treatment context, physiological trends, symptoms, uncertainty, and multimodal review priority, consistent with cautious interpretations of healthcare digital twins using wearable data [29].

### 2.5. Agentic AI for Clinical Workflow Orchestration

Agentic AI refers to systems composed of specialized agents that perform bounded tasks, coordinate outputs, and support workflow execution. In clinical sensing, this modularity is useful because imaging, wearable, respiratory, treatment-context, and explanation tasks require different quality checks, uncertainty rules, and safety boundaries.

Recent healthcare AI-agent reviews emphasize that the field remains early and requires clear definitions, rigorous evaluation, human oversight, and safety constraints [30,31]. OncoSense-Agent follows this principle by implementing bounded agents for review-priority support rather than autonomous diagnosis.

### 2.6. Literature Gap Analysis and Architecture Requirements

The architecture was derived from a structured analysis of seven areas: CT organ-at-risk segmentation, wearable oncology monitoring, respiratory remote monitoring, multimodal biomedical AI, digital patient-state modeling, explainable clinical AI, and agentic AI for healthcare workflows. The aim was not a formal systematic review, but translation of recurring limitations into sensing-system requirements. Please see Table 1 for the full details on our analysis.

The recurring gaps are consistent across modalities: CT segmentation requires reliability estimation [4,6,7]; wearable monitoring requires handling of missingness, adherence, and device variability [15,16,17,18,19]; respiratory signals require contextual interpretation with treatment phase and comorbidities [22,23,24,25,26,27]; and clinical multimodal AI requires explainability, auditability, privacy safeguards, and human oversight [28,29,30,32,33].

These gaps motivate a design principle: each agent addresses a specific sensing-system requirement. CT uncertainty motivates imaging reliability assessment, wearable noise motivates signal-quality scoring, respiratory ambiguity motivates pneumology-oriented review, fragmented data motivate fusion, clinical trust motivates explanation, and sensitive health data motivate governance.

**Figure 1:**
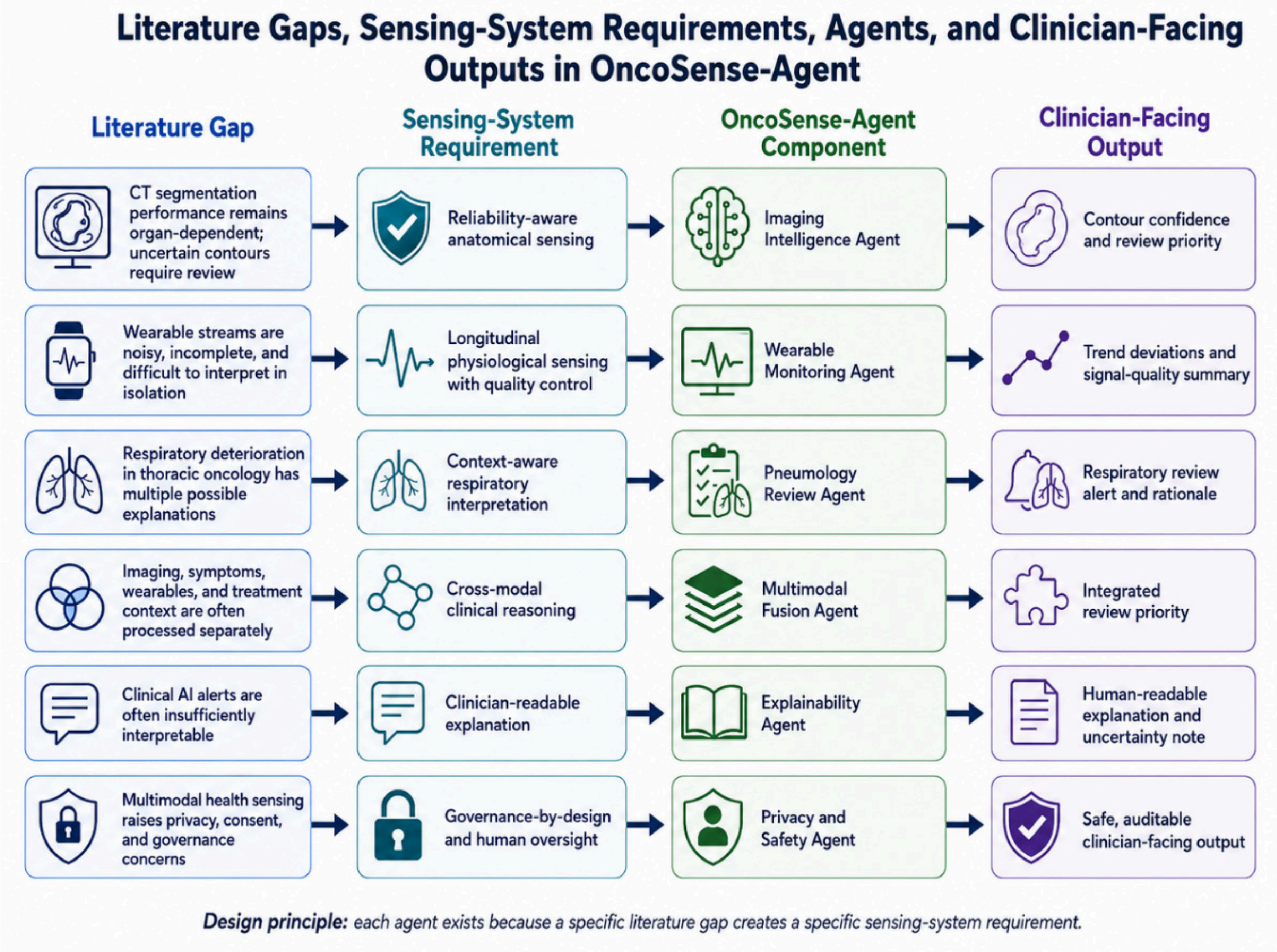
Literature gaps, sensing-system requirements, OncoSense-Agent components, and clinician-facing outputs. The figure summarizes the design principle used to derive the proposed architecture: each agent addresses a specific gap identified in prior literature and translates it into a sensing-system requirement and a clinically reviewable output.

Figure 1 summarizes how the literature gaps identified above are translated into sensing-system requirements, specialized OncoSense-Agent components, and clinician-facing outputs.

### 2.7. Closest Clinical Comparators: Oncology Remote Monitoring, Wearables, and Radiotherapy AI

The most clinically relevant comparators (see Table 2) are oncology symptom-monitoring systems, wearable-enabled oncology monitoring, lung-cancer digital health frameworks, multimodal cancer remote patient-monitoring models, and radiotherapy digital-twin or toxicity-prediction approaches. These systems address patient monitoring, longitudinal data, treatment toxicity, or radiotherapy context, but usually focus on one part of the workflow rather than reliability-scored CT, wearable, respiratory, and treatment-context fusion [16,17,19,34,35,36,37,38].

The comparison suggests that OncoSense-Agent is closest to oncology remote monitoring and radiotherapy digital-twin research, but differs by combining anatomical CT reliability, longitudinal physiological monitoring, respiratory symptom interpretation, treatment context, and explicit human-in-the-loop uncertainty escalation in one workflow.

### 2.8. Broader Agentic and Multimodal Healthcare AI Landscape

A second comparison is useful because OncoSense-Agent also belongs to the emerging family of agentic and multimodal healthcare systems. Table 3 compares OncoSense-Agent with recent frameworks that use multi-agent orchestration, multimodal inputs, dynamic routing, knowledge-guided reasoning, benchmark evaluation, or enterprise-scale clinical data infrastructure. This comparison is intended to clarify architectural positioning rather than claim superiority over systems developed for different clinical domains [31,39,40,41,42,43,44].

The broader landscape reinforces two points: healthcare agents require bounded roles, evaluation, safety constraints, and human oversight [30,31,43], and the compared systems do not explicitly address the same combination of CT-derived lung reliability, continuous physiological monitoring, respiratory review, treatment context, and Safety Gate escalation for thoracic oncology surveillance.

## 3. Materials and Methods

### 3.1. Prototype Validation Design

OncoSense-Agent was evaluated through a lung-focused prototype validation designed to test whether CT-derived anatomical evidence can be combined with longitudinal physiological, respiratory, symptom, and treatment-context information through a reliability-aware agentic workflow. The evaluation was not intended to establish diagnostic accuracy or prospective clinical benefit. Instead, it was designed to evaluate architecture behavior, agent communication, reliability scoring, Safety Gate escalation, and the difference between CT-only interpretation and multimodal review-priority generation.

The validation used a hybrid design. Real CT segmentation metrics were used as the imaging anchor, while wearable, respiratory, patient-reported, and treatment-context signals were introduced as deterministic overlays. The primary validation denominator was 139 real patients with complete bilateral lung CT metrics. This lung-focused scope was chosen to align the prototype with thoracic oncology and pneumology-oriented monitoring, where respiratory deterioration may occur even when CT-derived anatomical evidence appears stable.

### 3.2. Real CT Segmentation Anchor

The imaging component was based on a real nnU-Net v2 3D thoracic segmentation evaluation. Although the original evaluation included 15 organs at risk, the proof-of-concept was intentionally restricted to left and right lung structures. This restriction preserved domain coherence with the pneumology use case and avoided over-extending the prototype beyond its intended respiratory-monitoring scope.

The source CT evaluation provided the real anatomical evidence used by the Imaging Reliability Agent. It documented a 3D full-resolution nnU-Net v2 model evaluated on a locked thoracic test subset, with 159 evaluable thoracic cases derived from a larger private CT database containing 6719 CT series from 3117 patients. Evaluation metrics (see Table 4) were calculated at the patient level on 3D binary segmentations and included Dice, IoU, HD95, precision, recall, F1, and false-positive risk, following established segmentation-evaluation practice [45]. The evaluated model used a 160 × 128 × 80 voxel input patch, resampled spacing of 3.00 × 1.07 × 1.07 mm, CT intensity normalization with HU clipping to 0–170 HU, batch size 3, and standard nnU-Net augmentation. These details are reported to make clear that the CT anchor was derived from a real segmentation evaluation rather than from synthetic imaging values.

The CT Anchor Store included lung-level segmentation metrics such as Dice, IoU, precision, recall, and false-positive risk. The left lung showed Dice = 0.652, IoU = 0.506, precision = 0.526, and recall = 0.940. The right lung showed Dice = 0.711, IoU = 0.572, precision = 0.591, and recall = 0.954. These values indicate high recall but moderate precision, suggesting that the model generally captures lung regions but may still generate boundary false positives. Therefore, CT contours were treated as reliability-scored anatomical sensing evidence rather than automatically accepted ground truth.

### 3.3. Synthetic Multimodal Overlay Generation

For each patient in the lung-focused cohort, deterministic overlays were generated to represent wearable physiology, respiratory status, patient-reported symptoms, functional assessment, and treatment context. These variables (see Table 5) were not prospectively collected clinical monitoring data. They were generated to test how the architecture behaves under controlled and reproducible multimodal scenarios.

The synthetic overlays included oxygen saturation, heart rate, activity level, sleep duration, respiratory rate, dyspnea, cough, mMRC score, CAT score, six-minute walk test distance, treatment phase, radiotherapy dose context, and comorbidity status. Scenario templates represented stable monitoring, physiological decline, concurrent chemoradiotherapy risk, post-radiotherapy recovery, and COPD-related respiratory deterioration. This approach preserved real CT segmentation variability while allowing deterministic stress-testing of agent behavior under multimodal signal agreement and disagreement.

For reproducibility, the overlays were generated from fixed scenario templates rather than from a trained predictive model. Each template defined baseline-to-current changes in oxygenation, activity, heart rate, respiratory burden, functional status, and treatment context. Expected review-priority labels were therefore scenario labels used to test workflow behavior, not clinical outcome labels. This distinction is important because the proof-of-concept evaluates whether the architecture responds coherently to controlled patient-state patterns rather than measuring diagnostic accuracy.

### 3.4. Implemented Agentic Workflow

The implemented proof-of-concept included six bounded software agents: Imaging Reliability, Wearable Monitoring, Respiratory Review, Treatment Context, Multimodal Fusion, and Explainability. These agents were implemented as task-specific workflow modules rather than autonomous clinical decision-makers. Each agent had a defined input schema, processing mechanism, output schema, reliability logic, and safety boundary.

**Figure 2:**
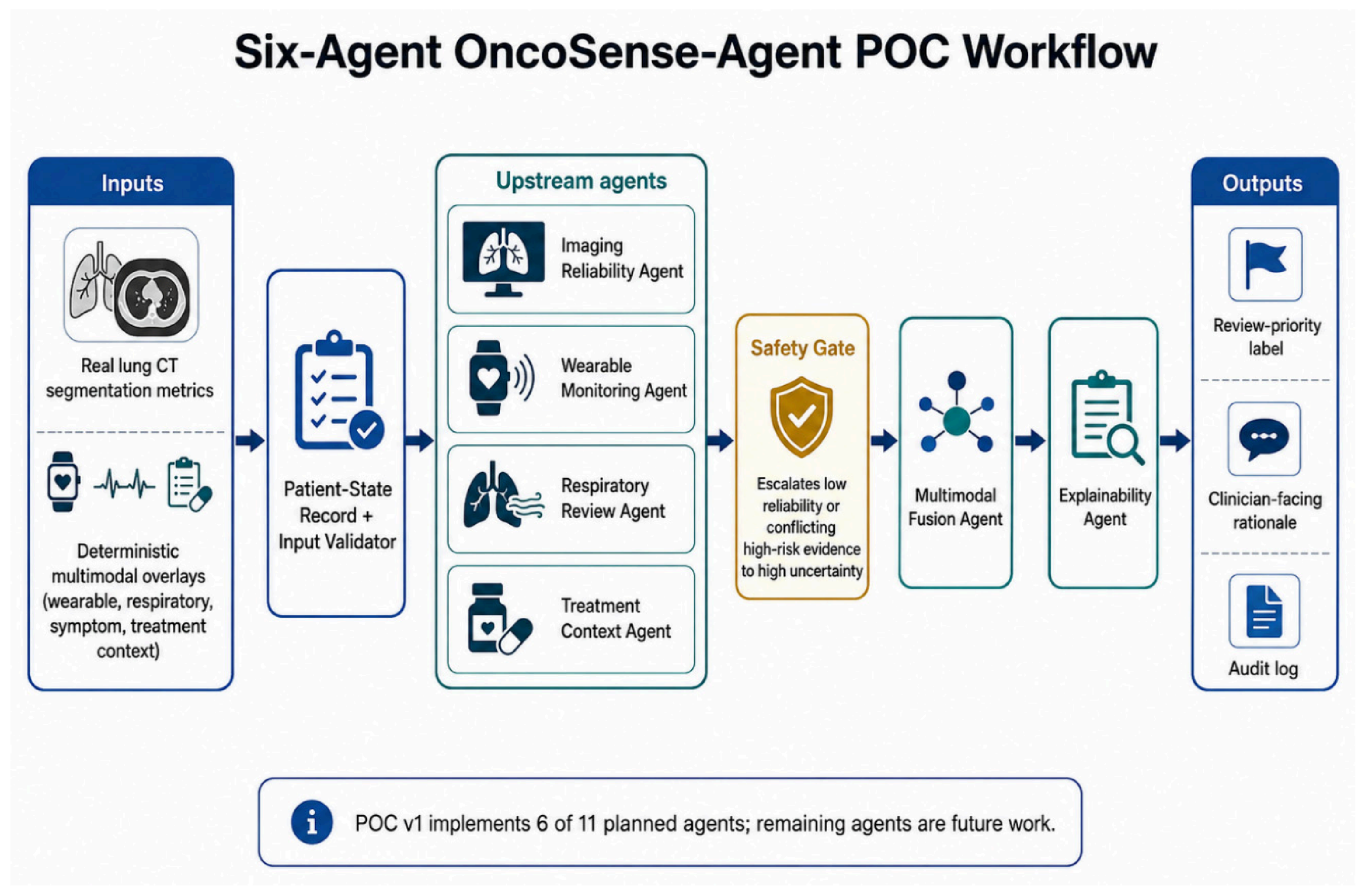
Six-agent workflow implemented in OncoSense-Agent POC v1. Real lung CT metrics and deterministic multimodal overlays form a validated patient-state record processed by four upstream agents: the Safety Gate, Multimodal Fusion, and Explainability. Outputs are the review-priority label, clinician-facing rationale, and audit log. POC v1 implements six of eleven planned agents; the remaining agents are future work.

Figure 2 provides a workflow-level view of the six implemented bounded agents and shows how real CT metrics and deterministic multimodal overlays are transformed into clinician-facing review-priority outputs. The figure complements the broader technical architecture by emphasizing execution order, Safety Gate placement, and auditable output generation within POC v1.

The implemented POC v1 operationalizes six of the eleven agents defined in the complete OncoSense-Agent architecture: Imaging Reliability, Wearable Monitoring, Respiratory Review, Treatment Context, Multimodal Fusion, and Explainability. The remaining five agents—Symptom Interpretation, Anomaly Detection, Dynamic Patient-State Modeling, Privacy and Safety Governance, and Clinical Reporting—are retained as specifications in the broader architecture but were not implemented in the current prototype. This scoping decision was intentional. The objective of POC v1 was to evaluate the core architectural claim: whether reliability-aware agentic fusion can convert real CT segmentation evidence and deterministic multimodal overlays into auditable review-priority outputs. Integration of the remaining agents is therefore treated as future work rather than an unmet component of the present validation.

### 3.5. Technical Architecture of the POC Implementation

**Figure 3:**
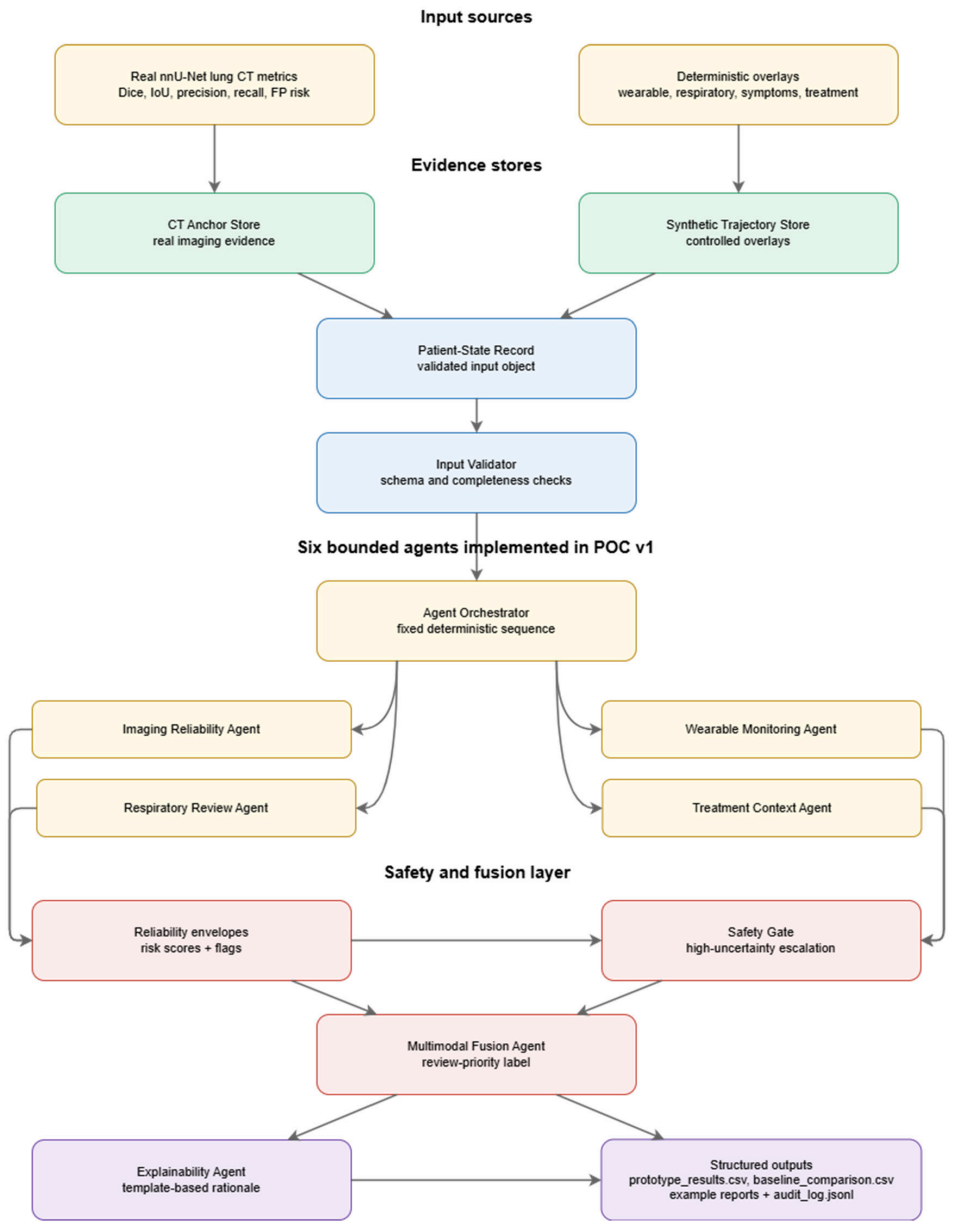
Technical architecture of the OncoSense-Agent POC implementation. Real lung CT segmentation metrics are loaded into the CT Anchor Store, while deterministic wearable, respiratory, symptom, and treatment-context overlays are loaded into the Synthetic Trajectory Store. Validated patient-state records are processed by six implemented bounded agents: Imaging Reliability, Wearable Monitoring, Respiratory Review, Treatment Context, Multimodal Fusion, and Explainability. The Safety Gate converts low reliability or conflicting high-risk evidence into auditable high-uncertainty review states. The pipeline produces structured outputs, including per-patient prototype results, baseline-comparison results, example clinician-facing reports, and an audit log. The remaining five agents from the full eleven-agent architecture are specified for future integration and were not implemented in POC v1.

Figure 3 summarizes the technical architecture of the implemented proof-of-concept. The workflow separates real imaging evidence from deterministic non-imaging overlays. Real lung CT segmentation metrics from the nnU-Net evaluation are loaded into a CT Anchor Store, while deterministic wearable, respiratory, symptom, and treatment-context overlays are loaded into a Synthetic Trajectory Store. Each patient-state record is validated and then processed by the six implemented bounded agents. The upstream agents generate modality-specific risk and reliability envelopes, which are passed to the Safety Gate and Multimodal Fusion Agent. The final output consists of a review-priority label, a clinician-facing explanation, and an audit record containing intermediate agent outputs. This implementation is deterministic and reproducible, but it does not perform live sensor ingestion, real-time nnU-Net inference, autonomous diagnosis, or treatment adaptation.

The implementation used a lightweight Python-based stack for deterministic execution and post hoc inspection. Tabular patient-state records were represented as CSV or JSON-like objects, and agent outputs were stored as structured messages containing agent name, score, reliability, categorical label, flags, explanation, and safety boundary. The output artifacts included per-patient prototype results, baseline-comparison tables, example clinician-facing reports, and JSONL audit logs. This structure was selected to make every intermediate decision traceable while keeping the POC independent of live clinical systems.

### 3.6. Technical Framework and Technology Stack

This subsection defines the implementation stack used for the current proof-of-concept and the recommended evolution path for a deployment-ready version. The implemented stack prioritizes transparency, deterministic behavior, and reproducibility over infrastructure complexity. This choice is intentional: the objective of POC v1 is to provide reviewer-facing prototype validation of reliability-aware review-priority generation, not to claim production deployment or real-time clinical integration.

#### 3.6.1. POC v1 Stack (Implemented Scope)

The implemented POC v1 was designed as a lightweight research prototype that can be executed locally and inspected through explicit artifacts. The stack, presented in Table 6, avoids unnecessary infrastructure layers while preserving typed agent contracts, deterministic scoring, auditable outputs, and reproducible reruns.

Operationally, POC v1 follows a command-line execution pattern. The pipeline can be run as a local research workflow, for example by executing a single orchestrator script. The data scope is limited to one static CT Anchor Store and deterministic multimodal trajectories. The security scope is file-level and research-environment based; production-grade identity, access management, consent enforcement, and GDPR/HIPAA controls are not implemented in the current prototype.

#### 3.6.2. Evolution Path Toward an Advanced Pre-Clinical Platform

A more advanced version should preserve the deterministic scoring path while adding clinical-grade interoperability, observability, governance, and deployment controls. Table 7 summarizes the recommended evolution path. These components are not part of the implemented POC v1 and are presented only as a future technical roadmap.

POC v1 intentionally avoids LLM-agent orchestration frameworks to preserve deterministic behavior, reproducibility, and auditable scoring. Graph-based frameworks such as LangGraph may be useful in future versions if the workflow evolves from fixed execution paths to dynamic stateful coordination with durable execution and human-in-the-loop checkpoints; LangChain-style agent abstractions may also be considered for higher-level tool-calling patterns when clinically appropriate [46,47].

The advanced technical roadmap is governed by four design priorities. First, the deterministic scoring path should be preserved for safety-critical review-priority decisions. Second, the Safety Gate should remain an independent, mandatory component with regression tests. Third, all input/output schemas and explanation templates should be versioned. Fourth, architecture claims should remain separated from validation claims: POC validation, controlled clinical pilot validation, and prospective outcome validation represent distinct evidence levels.

### 3.7. Reliability-Aware Fusion and Safety Gate

The Multimodal Fusion Agent [48] combined four evidence streams: imaging risk, wearable deviation, respiratory deterioration, and treatment-context risk. The baseline weighting was

Base Priority = 0.40 × Imaging Risk + 0.25 × Wearable Deviation + 0.20 × Respiratory Deterioration + 0.15 × Treatment Context Risk

The baseline fusion weights used in POC v1 were heuristic, expert-informed coefficients selected for interpretability and controlled stress-testing rather than optimized clinical parameters. Imaging received the largest weight because CT remains the dominant anatomical modality in thoracic oncology workflows, while wearable, respiratory, and treatment-context streams were assigned lower but clinically meaningful weights to allow longitudinal deterioration and treatment risk to modify CT-only interpretation. These coefficients should therefore be understood as initial POC weights, not validated clinical weights. Future work will calibrate and validate them against clinician-rated review priority, adjudicated clinical events, treatment-toxicity labels, and prospective patient outcomes using clinician consensus, regression-based optimization, calibration analysis, sensitivity analysis, and comparison with expert triage decisions.

The weighting reflects the central role of CT in thoracic oncology while allowing physiological and respiratory deterioration to modify review priority. A Safety Gate was added to avoid silent false reassurance when one or more modalities were unreliable. The Safety Gate escalated cases to a high-uncertainty review state when CT reliability was very low or when elevated priority was accompanied by insufficient overall reliability. This mechanism converts unreliable or conflicting data into an auditable clinician-review requirement rather than suppressing uncertainty.

Reliability was calculated using four dimensions: completeness, signal or model quality, temporal relevance, and source trustworthiness. This preserved the original reliability-scoring principle while making the calculation operational in the prototype.

R_m = w_c C_m + w_q Q_m + w_t T_m + w_s S_m

where R_m is the reliability score for modality m, C_m represents completeness, Q_m represents signal or model quality, T_m represents temporal relevance, and S_m represents source trustworthiness.

In the prototype implementation, the four reliability dimensions were operationalized differently by modality. For CT, quality was derived from segmentation metrics such as Dice, precision, recall, and false-positive risk. For wearable streams, completeness and quality reflected missingness, non-wear time, and signal plausibility. For respiratory and patient-reported inputs, completeness reflected the availability of dyspnea, cough, CAT, mMRC, and six-minute walk test variables. Treatment-context reliability was treated as high when derived from structured clinical documentation.

Review-priority thresholds were defined deterministically for the proof-of-concept. Low priority corresponded to a base priority below 0.30, moderate priority to values from 0.30 to 0.50, and high priority to values above 0.50. The Safety Gate escalated a case to high uncertainty when CT reliability was extremely low or when an elevated priority score was paired with insufficient mean reliability. This rule was included to prevent low-quality imaging or conflicting multimodal evidence from being interpreted as reassuring.

### 3.8. Baseline and Conflict-Testing Design

The baseline comparison was designed to test whether the reliability-aware agentic workflow provided behavior that differed from simpler strategies. Four baselines were defined: CT-only assessment, wearable-only assessment, rule-based thresholding, and non-agentic late fusion. CT-only assessment used imaging reliability and anatomical risk alone. Wearable-only assessment used physiological deviation alone. Rule-based thresholding applied fixed signal thresholds. Non-agentic late fusion averaged modality scores without per-agent reliability handling or Safety Gate escalation.

Two validation settings were considered. First, deterministic aligned overlays were used to verify expected behavior when all non-imaging signals pointed in the same direction. Second, adversarial conflict profiles were used to test behavior under realistic disagreement, including unreliable CT with respiratory deterioration, wearable device artifacts, stable imaging with worsening symptoms, and comorbidity-driven deterioration. This design avoids relying only on easy deterministic scenarios and directly tests the value of reliability-aware fusion under modality conflict.

### 3.9. Case-Based Validation Design

In addition to cohort-level analysis, three representative modeled case profiles were used to demonstrate how the agentic workflow behaves under clinically meaningful signal patterns. These cases were selected to illustrate different forms of architecture value: hidden deterioration despite acceptable CT metrics, wearable data-quality uncertainty, and complex conflict between improving imaging and worsening physiology. The cases used real lung CT metric anchors combined with deterministic multimodal overlays; they were not prospective patient outcome examples and were not used as the primary statistical evidence base. They were used to explain the decision logic of the proof-of-concept and to show why CT-only monitoring can be insufficient in thoracic oncology surveillance.

### 3.10. Ethics, Data Use, and Validation Boundaries

The CT segmentation metrics were derived from retrospective anonymized imaging data. No prospective wearable, respiratory, patient-reported, or treatment-monitoring data were collected for this proof-of-concept. Non-imaging modalities were deterministic synthetic overlays generated for prototype testing. Therefore, the evaluation should be interpreted as architecture-behavior validation, not as clinical diagnostic validation or evidence of prospective patient benefit.

The system output is limited to review-priority generation and explanation. It does not perform autonomous diagnosis, treatment adaptation, radiotherapy modification, or clinical intervention. All outputs are intended for human review in a clinician-supervised workflow.

### 3.11. Use of Generative Artificial Intelligence

Generative artificial intelligence tools were used to support language editing, manuscript structuring, and the preparation of schematic figure assets. GPT-5.5 via ChatGPT (OpenAI) was used to assist with English-language editing and the organization of the manuscript. The authors reviewed and revised all AI-assisted text to ensure accuracy, clarity, and consistency with the scientific content.

DALL·E 3 via ChatGPT (OpenAI) was used to assist in generating illustrative visual elements for Figure 1 and Figure 2, with the purpose of communicating the intended concepts and messages of the figures more clearly. The authors defined the scientific content, conceptual structure, terminology, labels, relationships between components, and intended interpretation of the figures. The generated outputs were subsequently reviewed, edited, corrected, and verified by the authors.

Figure 3 was completely drawn by the authors using diagrams.net (draw.io) and contains no AI-generated visual material.

## 4. Results: Cohort-Level and Case-Based POC Validation

This section presents the prototype-level validation of OncoSense-Agent. The evaluation combines a cohort-level analysis using 139 patients with complete bilateral lung CT metrics, baseline and conflict testing, and a three-patient case study designed to illustrate how the architecture changes CT-only interpretation through multimodal fusion. The 139-patient cohort provides the primary quantitative validation context, while the three patient cases are used as explanatory examples of decision logic.

The evaluation should be interpreted as architecture-behavior validation rather than clinical diagnostic validation. Real CT segmentation metrics were used as the imaging anchor, while wearable, respiratory, symptom, and treatment-context channels were introduced as deterministic overlays for controlled and reproducible testing. The three-patient case study is illustrative and is not used as the primary evidence base.

### 4.1. Cohort-Level Validation in 139 Patients

The lung-focused proof-of-concept was evaluated on 139 patients with complete bilateral lung CT segmentation metrics. The cohort was derived from the same locked thoracic nnU-Net evaluation and included real per-patient Dice, precision, recall, F1, IoU, HD95, and false-positive-risk values for left and right lung structures. Non-imaging channels were added as deterministic overlays for the same patient records, enabling controlled testing of multimodal fusion while preserving real imaging variability.

The validation cohort covered a broad CT reliability spectrum, with CT reliability ranging from 0.0177 to 0.8600, a mean reliability of 0.4065, and a median reliability of 0.4292. This heterogeneity allowed the Safety Gate and reliability-aware fusion mechanism to be tested under realistic segmentation-quality variation rather than only under ideal imaging conditions.

The source CT evaluation also documented design-relevant limitations, including domain shift between validation and locked-test performance, partial annotations for rare organs, repaired slice-order/orientation handling during DICOM conversion, and special handling of complete segmentation failures in HD95 computation. These limitations reinforce the architectural decision to treat CT-derived contours as reliability-scored sensing outputs rather than as automatically accepted anatomical truth and are consistent with broader cautions about overinterpreting biomedical image-analysis rankings or single aggregate metrics [49]. Consequently, the CT segmentation experiment serves as the real imaging anchor for the prototype, while the central contribution remains the reliability-aware agentic orchestration of imaging, physiological, respiratory, and treatment-context evidence.

Across the 139-patient cohort, detailed in Table 8, OncoSense-Agent changed review-priority assignment relative to CT-only assessment in 78 cases, corresponding to 56.1%. This result indicates that the multimodal workflow frequently modified anatomical interpretation when physiological, respiratory, treatment-context, or reliability evidence suggested a different review priority. The high divergence rate is expected in this prototype because CT provides episodic anatomical information, while wearable and respiratory overlays represent dynamic patient-state changes.

The proof-of-concept also produced conservative review behavior: 111/139 patients were assigned to either high-priority or high-uncertainty tiers. This should not be interpreted as disease prevalence or diagnostic performance. Instead, it reflects how the prototype behaves when deterministic monitoring overlays are used to stress-test escalation, uncertainty handling, and clinician-review prioritization.

### 4.2. Review-Priority Distribution

The final proof-of-concept outputs were grouped into four review-priority tiers: low, moderate, high, and high uncertainty. The high-uncertainty tier indicates that the system identified a need for manual review because risk was clinically relevant but confidence was limited by imaging quality, modality conflict, or insufficient reliability.

The large proportion of high-priority and high-uncertainty outputs (see Table 9) demonstrates that the prototype behaves conservatively in a thoracic oncology monitoring setting. This is appropriate for a human-in-the-loop review-priority system, where the objective is not autonomous diagnosis but prioritization of patients who may require clinical attention. Importantly, high uncertainty is not equivalent to high disease probability; it represents an auditable request for human review when data quality, signal conflict, or low reliability prevents confident interpretation.

Direct Safety Gate escalations and high-uncertainty final labels were treated as related but distinct quantities. Direct Safety Gate escalations represent explicit rule-triggered uncertainty escalation, whereas high-uncertainty final labels also include final uncertainty states arising from modality conflict or low-confidence multimodal evidence.

### 4.3. CT-Only Versus Multimodal Review-Priority Divergence

CT-only assessment uses imaging reliability and anatomical-risk estimates as the main basis for review priority. In contrast, OncoSense-Agent integrates CT reliability with wearable physiology, respiratory deterioration, treatment context, and uncertainty logic. The comparison therefore tests whether multimodal orchestration modifies review-priority decisions beyond imaging alone.

Across the cohort, OncoSense-Agent changed the CT-only review-priority assignment in 78/139 patients. This 56.1% divergence shows that CT-only evaluation and multimodal patient-state evaluation are not equivalent. CT-only assessment can miss deterioration when anatomy appears stable, but physiology worsens, and it can also over- or under-weight imaging findings when segmentation reliability is limited.

The observed divergence supports the central purpose of the architecture: CT-derived contours should function as one reliability-scored sensing stream within a broader clinical monitoring workflow, not as the sole determinant of patient state.

### 4.4. Safety Gate Behavior Across CT Quality Tiers

The Safety Gate was designed to prevent unreliable data from producing silent false reassurance. It escalates cases for human review when CT reliability is very low or when elevated review priority is accompanied by insufficient overall confidence.

As seen in Table 10, Safety Gate activation increased as CT quality decreased. This indicates that the system was sensitive to segmentation uncertainty and did not blindly fuse low-quality imaging outputs with other modalities.

Safety Gate activation increased from 15.8% in excellent CT cases to 60.0% in very poor CT cases. Management divergence also increased from 42.1% in excellent CT cases to 80.0% in very poor CT cases. This pattern supports the intended behavior of the architecture: when CT evidence becomes less reliable, the system increasingly depends on multimodal context and escalates uncertainty for human review.

This behavior is clinically important because segmentation failure or poor contour reliability should not be interpreted as normality. Instead, uncertainty should be surfaced as a review requirement. At the same time, Safety Gate activation did not reach 100% even in the very poor CT tier, suggesting that the system remained selective rather than automatically escalating every low-quality imaging case.

### 4.5. Baseline and Conflict Testing

The proof-of-concept was compared with CT-only, wearable-only, rule-based threshold, and non-agentic late-fusion baselines (see Table 11). In deterministic aligned overlays, simple strategies could match the expected scenario labels because the synthetic signals were intentionally separated by scenario. Therefore, deterministic classification performance alone was not treated as evidence of clinical superiority. The more informative evaluation was the conflict-testing setting, where modalities deliberately disagreed.

The adversarial conflict cases are particularly important because they test the architecture under conditions closer to real clinical monitoring, where CT, wearable, respiratory, and treatment-context evidence may disagree. In these cases, imaging-only logic tended to under-escalate because CT cannot represent respiratory deterioration or device artifacts, while simple rule-based approaches could overreact to single abnormal signals. OncoSense-Agent provided intermediate behavior by combining modality-specific scores, reliability estimates, Safety Gate logic, and clinician-facing explanation.

### 4.6. Three-Patient Case Study: Mechanistic Validation of Architecture Value

To make the cohort-level behavior clinically interpretable, three representative patient cases, with details presented in Table 12, were analyzed in detail. These cases were selected to demonstrate different limitations of CT-only monitoring and different strengths of the agentic workflow. They are illustrative examples of decision logic, not the primary quantitative validation evidence.

Together, the cases show that OncoSense-Agent does not simply increase alerts. It changes review priority based on the relationship between anatomy, physiology, respiratory symptoms, treatment context, and data reliability.

### 4.7. Case 1—RC-047: Stable CT but Worsening Respiratory and Wearable Signals

RC-047 represents a paradoxically concerning patient profile. The patient was modeled as a 68-year-old male with stage IIIB lung cancer during concurrent chemoradiotherapy. CT-only assessment produced a moderate-risk interpretation because left-lung segmentation metrics were acceptable: Dice = 0.652, precision = 0.526, recall = 0.940, and IoU = 0.506. The CT quality score was 0.7173, corresponding to an anatomical-risk estimate of 0.2827. Based on imaging alone, the case could be interpreted as mild anatomical risk with routine or moderately intensified follow-up.

The multimodal workflow changed this interpretation. The Wearable Monitoring Agent detected a decrease in SpO2 from 95% to 91%, a heart-rate increase from 72 to 88 bpm, activity decline from 3500 to 1800 steps per day, and reduced sleep duration. The Respiratory Review Agent detected a respiratory-rate increase, worsening dyspnea, increased cough, higher CAT score, and reduced six-minute walk distance. The Treatment Context Agent added risk from ongoing concurrent chemoradiotherapy.

The Multimodal Fusion Agent integrated these signals and escalated the case to high review priority. The key finding is that CT-derived anatomy alone did not capture the full patient state. Longitudinal physiological and respiratory signals revealed deterioration that would not be visible from anatomical information alone.

RC-047 demonstrates that stable or moderately concerning CT evidence should not automatically imply patient stability. In thoracic oncology, worsening oxygenation, activity, dyspnea, cough, and functional capacity can justify earlier review even when CT anatomy does not appear severely alarming.

### 4.8. Case 2—RC-102: Good CT Response but Wearable Data-Quality Uncertainty

RC-102 represents a noisy-data scenario. The patient was modeled as a 72-year-old female after hypofractionated radiotherapy. CT-only assessment suggested low risk because right-lung segmentation metrics were stronger than the left-lung metrics: Dice = 0.711, precision = 0.591, recall = 0.954, and IoU = 0.572. The CT quality score was 0.7597, and anatomical risk was 0.2403, below the low-risk threshold in the CT-only logic.

The wearable overlay introduced potentially concerning physiological signals, including SpO2 decrease from 94% to 89%, heart-rate increase from 68 to 82 bpm, and activity decline from 4200 to 2100 steps per day. However, the case also included a wearable device transition, reducing confidence in the physiological signal. The Wearable Monitoring Agent therefore did not treat the abnormal values as fully reliable evidence.

OncoSense-Agent assigned moderate review priority with a data-quality flag. This output differs from both simple escalation and simple dismissal. The system recognized the potential clinical relevance of the abnormal wearable values but recommended verification rather than immediate high-priority escalation.

RC-102 demonstrates the value of reliability-aware monitoring. The system does not blindly amplify abnormal sensor readings. Instead, it evaluates signal reliability, detects possible device-related uncertainty, and generates a cautious review recommendation.

### 4.9. Case 3—RC-203: Imaging Acceptable but Patient-State Deteriorating

RC-203 represents a complex-conflict scenario. The patient was modeled as a 65-year-old male with stage IIIA small-cell lung cancer during concurrent chemoradiotherapy. CT-only assessment produced a moderate-risk interpretation because the imaging evidence was not strongly alarming. The left-lung Dice score was 0.652, precision was 0.526, recall was 0.940, and the CT quality score was 0.7173. In an imaging-only workflow, the patient could be interpreted as responding to therapy or remaining anatomically stable.

The multimodal workflow identified a different pattern. The Wearable Monitoring Agent detected SpO2 decline from 93% to 88%, heart-rate increase from 70 to 96 bpm, activity collapse from 5000 to 1200 steps per day, and sleep reduction from 7 h to 2.5 h per night. The Respiratory Review Agent and Treatment Context Agent further increased concern because of respiratory deterioration, active treatment, and comorbidity-related vulnerability.

OncoSense-Agent escalated the case to high review priority with a comorbidity interaction flag. This case demonstrates that apparent imaging stability or response does not necessarily imply safe patient status. Physiological reserve, respiratory symptoms, and treatment toxicity can deteriorate before or independently of clear anatomical progression.

RC-203 demonstrates the strongest clinical value of the architecture: it can identify high-risk patient-state deterioration when imaging alone appears acceptable.

### 4.10. Cross-Case Interpretation

Across the three cases, OncoSense-Agent added value in three distinct ways. For details, we refer the reader to Table 13.

In RC-047, the system escalated review priority because physiology and respiratory symptoms worsened despite non-alarming CT findings. In RC-102, the system avoided both false reassurance and overreaction by identifying wearable data-quality uncertainty. In RC-203, the system detected multimodal deterioration and comorbidity-related risk despite acceptable imaging. The case-study pattern supports the central claim that CT-only surveillance is incomplete for longitudinal thoracic oncology monitoring.

### 4.11. Summary of Results

The 139-patient cohort shows that OncoSense-Agent frequently changes review-priority assignments compared with CT-only assessment. The divergence rate of 56.1% indicates that multimodal patient-state modeling is not redundant with imaging-only evaluation. The Safety Gate analysis further shows that the system responds progressively to declining CT quality, transforming low reliability into auditable review requirements rather than silent false reassurance. Baseline and adversarial conflict testing further support the need for reliability-aware orchestration because CT-only and simpler fusion strategies cannot consistently represent modality conflict, signal uncertainty, and explainable escalation behavior.

The three-patient case study explains why this matters clinically. CT-only monitoring can under-call risk when anatomy appears stable, but physiology deteriorates, can miss data-quality problems in wearable monitoring, and can fail to represent treatment context or comorbidity-driven deterioration. OncoSense-Agent addresses these limitations by combining imaging reliability, wearable trends, respiratory symptoms, treatment context, and explainability in a bounded human-in-the-loop workflow.

These results do not establish clinical effectiveness or diagnostic accuracy. They demonstrate that the implemented proof-of-concept is operational, auditable, and capable of producing clinically interpretable review-priority outputs under controlled validation conditions. Prospective validation with real wearable, respiratory, treatment, and outcome data remains required before clinical deployment.

## 5. Discussion

### 5.1. Main Findings

This proof-of-concept evaluates OncoSense-Agent as an implemented workflow for reliability-aware multimodal review-priority generation. The central quantitative finding is that multimodal fusion changed review-priority assignment relative to CT-only assessment in 78/139 patients (56.1%), showing that CT-only interpretation and multimodal patient-state assessment are not equivalent in this thoracic oncology monitoring setting.

The cohort-level results also show conservative behavior under uncertainty: 111/139 cases were assigned to high-priority or high-uncertainty tiers, and direct Safety Gate escalation occurred in 41/139 cases. These values represent prototype review-priority behavior under deterministic overlays, not disease prevalence or diagnostic accuracy.

The three illustrative cases clarify the mechanism: respiratory and wearable deterioration can justify escalation despite non-alarming CT, abnormal wearable values require reliability checks, and treatment context or comorbidity can reveal risk even when imaging appears acceptable.

### 5.2. Architecture Value of Reliability-Aware Fusion

The main value of OncoSense-Agent is reliability-aware orchestration, not replacement of individual component models. CT segmentation, wearable monitoring, respiratory questionnaires, and explainable reporting are established directions; the contribution is their coordination into a clinician-facing review-priority workflow.

This is important in thoracic oncology because CT contours provide episodic anatomical evidence, while wearable and respiratory streams provide dynamic but noisy information. The fusion mechanism treats each stream as partial evidence with modality-specific reliability before assigning review priority.

The Safety Gate is the core safety mechanism: low reliability or conflicting high-risk evidence becomes an explicit human-review requirement rather than a hidden uncertainty inside a fused score.

### 5.3. From Six-Agent POC to the Full OncoSense-Agent Architecture

The implemented proof-of-concept used six bounded agents because this scope was sufficient to test the core workflow: imaging reliability, wearable monitoring, respiratory review, treatment-context reasoning, multimodal fusion, and explanation. This six-agent implementation should be distinguished from the broader eleven-agent OncoSense-Agent architecture. The five agents not implemented in POC v1—Symptom Interpretation, Anomaly Detection, Dynamic Patient-State Modeling, Privacy and Safety Governance, and Clinical Reporting—remain specified as extension components for the complete architecture. This distinction is important because the current validation is intended to test reliability-aware review-priority generation, not full deployment-scale clinical reporting, autonomous anomaly detection, or end-to-end privacy-governance automation.

This staged design is intentional. A broad architecture can be proposed, but only the implemented lung-focused core should be treated as the validated component in the present study. Future versions can extend the same reliability-aware orchestration logic to additional organs at risk, real-time streaming data, external clinical systems, and governance agents once each component has been validated.

### 5.4. Clinical Workflow Implications

The intended output of OncoSense-Agent is not a diagnosis, prognosis, treatment adaptation, or radiotherapy modification. The output is a review-priority category accompanied by reliability scores, contributing evidence, uncertainty flags, and a clinician-facing explanation. This makes the architecture suitable for human-in-the-loop monitoring workflows where the system helps prioritize attention but does not replace physician judgment.

In a clinical workflow, the system could support periodic review during thoracic radiotherapy or post-treatment surveillance. A low-priority output would support routine monitoring. A moderate-priority output would suggest scheduled review or repeated measurement. A high-priority output would suggest earlier oncology or pneumology review. A high-uncertainty output would indicate that the available data are insufficiently reliable and require verification or clinician interpretation before further action.

### 5.5. Positioning Against Existing Research Directions

Section 2.7 and Section 2.8 position OncoSense-Agent against close clinical comparators and broader agentic healthcare AI systems. Existing oncology RPM, wearable monitoring, digital lung-cancer tools, radiotherapy digital twins, and toxicity-prediction models show that symptoms, wearables, imaging, dose, and clinical context can support cancer monitoring, but they usually address only part of the sensing workflow [16,17,19,34,35,36,37,38].

Broader agentic systems demonstrate progress in dashboards, tool-using treatment-planning agents, specialist routing, knowledge-guided reasoning, and enterprise-scale AI platforms [31,39,40,41,42,43,44]. However, they are usually not focused on CT-guided respiratory monitoring in thoracic oncology or explicit Safety Gate escalation.

OncoSense-Agent therefore occupies a narrower niche: reliability-aware multimodal review-priority generation for CT-guided respiratory monitoring in oncology. It does not claim novelty in nnU-Net segmentation, wearable analytics, or questionnaires; the contribution is auditable orchestration under human oversight.

## 6. Limitations and Future Work

### 6.1. Prototype Validation Boundaries

The main limitation is the semi-synthetic nature of the proof-of-concept. The imaging anchor was derived from real lung CT segmentation metrics, but wearable, respiratory, symptom, and treatment-context variables were deterministic overlays rather than prospectively collected longitudinal patient data. The outputs therefore demonstrate architecture behavior, reproducibility, agent communication, reliability-aware fusion, and Safety Gate logic under controlled scenarios; they do not establish diagnostic accuracy, treatment benefit, clinical effectiveness, or patient-outcome improvement.

Additional limitations are the lung-focused scope, the six-agent implementation, and the absence of deployment-scale integration. The current validation does not yet cover multi-organ reasoning, live EHR integration, privacy-governance automation, clinical-report generation, or real-world missing/conflicting data streams. Prospective validation with real multimodal data is required before clinical deployment.

### 6.2. Prospective Validation Roadmap

Future work should collect real wearable, respiratory, patient-reported, treatment-context, and outcome data in a prospective thoracic oncology or radiotherapy cohort. Such a pilot should evaluate recruitment, adherence, missingness, signal quality, patient burden, clinician review time, alert burden, and acceptability. Review-priority outputs should be compared with clinician-rated priority and clinically relevant outcomes such as unscheduled visits, pneumology referral, treatment interruption, documented toxicity, or radiological reassessment.

The reliability and fusion weights should also be calibrated in future studies. The current sensitivity analysis evaluates whether POC behavior remains stable under weight perturbation, but it does not replace calibration against clinician-rated priority, expert triage decisions, and patient outcomes. Explainability should be evaluated separately through clinician assessments of clarity, usefulness, uncertainty communication, and workflow fit.

### 6.3. Extension to Multiorgan and Real-Time Monitoring

The next technical step is to extend the lung-focused proof-of-concept to additional organs at risk and to real multimodal data streams. This includes esophagus, heart, spinal cord, and other thoracic or abdominal organs when clinically relevant. Multiorgan extension should be performed cautiously because the segmentation performance and annotation quality vary substantially by structure. Each organ should therefore have its own reliability model, review threshold, and validation evidence.

A later deployment version should integrate DICOM and RTSTRUCT ingestion, wearable APIs, respiratory home monitoring, structured questionnaires, and EHR context through secure interoperability standards such as DICOM and FHIR [50,51]. Privacy governance, audit logging, consent verification, and blocked-action rules should be implemented before any clinical pilot. The system should remain clinician-supervised, with explicit restrictions against autonomous diagnosis, treatment recommendation, or radiotherapy adaptation.

## 7. Conclusions

OncoSense-Agent is a reliability-aware agentic multimodal sensing architecture for CT-guided respiratory monitoring in oncology care. The system links CT-derived anatomical evidence with wearable physiology, respiratory and functional measures, symptoms, treatment context, and clinician-facing explanations. The implemented lung-focused proof-of-concept used six bounded software agents and real lung CT segmentation metrics derived from a 3D nnU-Net v2 evaluation in 139 patients with complete bilateral lung CT data, complemented by deterministic multimodal overlays for controlled validation.

The proof-of-concept changed review-priority assignment relative to CT-only assessment in 78/139 cases and demonstrated conservative uncertainty handling through high-priority/high-uncertainty assignments and direct Safety Gate escalations. The modeled case-study examples illustrate why this matters: CT-only monitoring can miss hidden respiratory deterioration, fail to account for wearable data quality, or underestimate treatment-context and comorbidity-driven risk.

Accordingly, the results should be interpreted as methodological and prototype-level evidence: the workflow demonstrates feasible orchestration, auditability, and reliability-aware review-priority generation, while prospective validation with real wearable, respiratory, treatment, and outcome data remains a requirement before clinical deployment.

## Figures and Tables

**Table 1:** Literature-derived requirements and corresponding OncoSense-Agent architectural components.

Literature Area	Evidence from Prior Work	Remaining Gap	Sensing-System Requirement	OncoSense-Agent Response
CT organ-at-risk segmentation	nnU-Net, TotalSegmentator, and MedSAM show strong progress in biomedical and CT segmentation [4,6,7].	Segmentation remains organ-dependent and clinically context-sensitive.	Reliability-aware anatomical sensing.	Imaging Intelligence Agent with contour confidence, anatomical plausibility checks, organ-name harmonization, and clinician review priority.
Wearable oncology monitoring	Wearables can capture activity, sleep, heart rate, HRV, oxygen saturation, and recovery patterns [15,16].	Implementation remains limited by missingness, adherence, comfort, device variability, and uncertain interpretation [16,17,18,19].	Longitudinal physiological sensing with quality control.	Wearable Monitoring Agent with baseline normalization, missingness detection, non-wear detection, adherence monitoring, and signal-quality scoring.
Respiratory remote monitoring	Remote respiratory monitoring and COPD digital health studies support home/wearable follow-up [22,23]; 6MWT, mMRC, and CAT provide standardized functional and symptom measures [24,25,26].	Respiratory decline in thoracic oncology has competing causes and requires clinical context.	Context-aware respiratory interpretation.	Pneumology Review Agent linking SpO2, respiratory rate, activity, symptoms, spirometry/PEF, 6MWT, mMRC, CAT, CT lung context, comorbidities, and treatment phase.
Multimodal biomedical AI	Multimodal AI can combine imaging, signals, symptoms, and clinical variables [28].	Many systems lack workflow-level review prioritization, reliability assessment, and radiotherapy-specific context.	Cross-modal clinical reasoning.	Multimodal Fusion Agent combining anatomy, physiology, symptoms, treatment timing, and modality reliability.
Digital patient-state modeling	Wearable sensors can contribute to dynamic healthcare digital twins and patient-state representations [29].	Full biological digital twins remain difficult to validate clinically.	Pragmatic patient-state representation.	Dynamic Patient-State Agent representing anatomy, treatment, physiology, symptoms, uncertainty, reliability, and multimodal risk.
Explainable clinical AI	XAI is central to CDSS usability, transparency, and trust [32,33].	Alerts are often difficult to interpret or insufficiently actionable.	Clinician-readable explanation.	Explainability Agent generating rationale, uncertainty summary, contributing evidence, and suggested review target.
Agentic AI in healthcare	Agentic AI can support complex workflows but remains early and requires safety validation [30].	Autonomous medical agents raise safety, accountability, and governance concerns.	Human-in-the-loop orchestration.	Bounded agentic AI layer with clinician oversight and no autonomous diagnosis or treatment adaptation.
Privacy-aware health AI	Multimodal sensing uses sensitive imaging, physiological, symptom, and clinical data.	Integrated sensing increases privacy, consent, and auditability requirements.	Governance-by-design.	Privacy and Safety Agent enforcing consent, data minimization, access control, audit logs, blocked actions, and escalation rules.

**Table 2:** Closest clinical and technical comparators to OncoSense-Agent.

Comparator	Main Modalities	Clinical Focus	Contribution	Gap relative to OncoSense-Agent
ePRO/remote symptom monitoring	Patient-reported symptoms, alerts, clinician dashboard	Cancer treatment symptom surveillance	PRO-TECT and related studies support the value of structured remote symptom monitoring during cancer treatment [34].	Usually symptom-centered; does not include CT segmentation reliability, wearable physiology, respiratory functional metrics, or agentic Safety Gate logic.
Wearable oncology monitoring	Activity, steps, heart rate, sleep, sometimes SpO2/HRV	Treatment monitoring, function, rehabilitation, toxicity surveillance	Reviews and pilots show increasing use of wearables in oncology but highlight adherence, missingness, device variability, and workflow barriers [16,17,19].	Often device- or feasibility-centered; lacks CT-guided anatomical context and explicit uncertainty escalation.
Digital health across lung-cancer continuum	Wearables, apps, cardiopulmonary and activity data	Lung-cancer monitoring, survivorship, performance status	Lung-cancer-specific digital health reviews identify wearables for activity, pulmonary function, cardiopulmonary monitoring, and complication detection [35].	Provides clinical rationale but not a concrete reliability-weighted agentic fusion architecture.
Multimodal AI RPM in cancer care	Demographics, wearables, daily surveys, clinical events	Adverse-event risk forecasting during systemic therapy	A recent multimodal RPM framework used real-world asynchronous data and reported AUROC 0.70 for future adverse-event risk [36].	Predictive-model focused; does not use CT contour reliability or explicit Safety Gate escalation.
Adaptive radiotherapy digital twin	Per-fraction PET/CT, dose, radiomics, dosiomics, temporal modeling	NSCLC adaptive radiotherapy and toxicity early warning	COMPASS models evolving organ response from dense imaging/dose streams in NSCLC [37].	Very close to radiotherapy, but lacks wearable, respiratory, and PRO monitoring streams.
Multimodal radiotherapy toxicity prediction	CT, dose distributions, clinical metadata	Treatment toxicity prediction	Deep learning models show how imaging, dose, and clinical metadata can be fused for toxicity prediction [38].	Prediction model rather than bounded agentic review-priority orchestration with audit logs.

**Table 3:** Comparative landscape of OncoSense-Agent and recent multimodal or agentic healthcare AI frameworks.

System	Modalities	Agentic Orchestration	Fusion Strategy	Reliability/Confidence Scoring	Wearables	Oncology/Pneumology Focus	Key Differentiator
OncoSense-Agent	CT lung metrics, wearable physiology, respiratory/PRO variables, treatment context	Yes; six bounded prototype agents	Reliability-weighted fusion plus Safety Gate	Explicit per-modality reliability and high-uncertainty escalation	Yes	Yes; thoracic oncology/pneumology	Reliability-aware review-priority generation with CT-guided respiratory monitoring
Cerebra [39]	EHR, clinical notes, imaging	Yes; multidisciplinary AI board	Multi-agent synthesis into dashboard	Robustness to incomplete modalities, but no wearable reliability equation	No reported continuous wearable stream	No; dementia risk	Large-scale multimodal clinical decision support
FUAS-Agents [40]	MRI, patient profiles, treatment tools, guidelines	Yes; multimodal LLM agents	Tool-using planning workflow	Reflection/internal quality-control mechanism	No	No; focused ultrasound treatment planning	Safety via reflection and guideline/tool checking
MedRoute [41]	Medical text/images and specialist-agent outputs	Yes; specialist agents plus RL router	RL-based specialist routing and moderator	Implicit through routing/moderation	No	No; general diagnosis tasks	Adaptive specialist routing
XMedFusion [42]	Medical images, visual evidence, knowledge graph, reports	Yes; knowledge-guided agents	Knowledge-graph-guided reasoning	Transparency via evidence/verification loops	No	No; image interpretation/reporting	Knowledge-graph synthesis for transparency
AgentRx [43]	EHR time series, CXR, reports, patient summaries	Benchmarks single vs multi-agent systems	Compares agent strategies	Calibration and forecasting metrics	No	No; clinical forecasting benchmark	Cautions that naive multi-agent systems may underperform
Mayo Clinic Platform [44]	Multi-institutional EHR, imaging, labs, notes	Platform-level infrastructure, not primarily agentic	Enterprise data/model infrastructure	Platform governance and clinical risk modeling	Partial through clinical data streams	Broad healthcare	Operational scale and real-world clinical research infrastructure

**Table 4:** Lung-focused CT metrics used as the imaging anchor.

Metric	Left Lung	Right Lung	Interpretation
N	143	142	Number of evaluable organ-level cases
Dice	0.652	0.711	Segmentation overlap; moderate lung contour quality
IoU	0.506	0.572	Complementary overlap metric
Precision	0.526	0.591	Moderate boundary false-positive tendency
Recall	0.940	0.954	High sensitivity for lung-region capture
FP risk	0.100	0.110	False-positive risk used in reliability scoring

**Table 5:** Deterministic overlay variables used for prototype testing.

Domain	Variables	Purpose in the Prototype
Wearable physiology	SpO2, heart rate, activity, sleep, missingness, non-wear time	Baseline-normalized physiological deviation and sensor reliability
Respiratory status	Respiratory rate, dyspnea, cough, CAT, mMRC, 6MWT	Respiratory deterioration scoring and pneumology review support
Treatment context	RT phase, fraction number, dose-risk level, chemotherapy, COPD/comorbidity	Treatment-context risk and comorbidity-aware interpretation
Scenario templates	Stable, physiological decline, concurrent chemoRT, post-RT recovery, COPD exacerbation	Controlled testing of agreement, conflict, and uncertainty behavior

**Table 6:** Implemented technology stack for POC v1.

Stack Area	POC v1 Implementation	Rationale
Runtime and language	Python 3.11	Fast iteration, strong scientific-computing ecosystem, and reproducible script execution.
Agent contracts	Dataclasses or Pydantic-ready typed schemas	Explicit inputs and outputs for each bounded agent; supports future schema validation.
Numerical processing	NumPy and pandas	Reliable scoring, deterministic synthetic generation, and tabular cohort analysis.
Parallel execution	concurrent.futures/ThreadPoolExecutor, optional	Simple parallel execution for upstream Tier-1 agents without microservices.
Data persistence	Local CSV, JSON, and JSONL artifacts	Fully auditable file-based workflow for results, reports, and intermediate agent outputs.
Explainability output	Template-driven text generation	Deterministic clinician-facing rationales; no free-form autonomous generation.
Visualization	matplotlib	Manuscript figures for architecture, baseline comparison, and scenario summaries.
Evaluation metrics	scikit-learn-style metrics and custom scripts	Accuracy, sensitivity, specificity, PPV, F1, label-change rate, and conflict-testing summaries.
Quality controls	pytest, ruff, black, optional	Minimal but credible testing and code-quality baseline for supplementary reproducibility.
Reproducibility	Fixed seeds and pinned requirements	Deterministic reruns using fixed random seeds and a versioned dependency file.

**Table 7:** Recommended advanced stack for a pre-clinical or deployment-ready version.

Capability Target	Advanced Stack Recommendation	Purpose
API and service boundary	FastAPI service layer for orchestrator and agent endpoints	Defines versioned service contracts for agent execution.
Workflow orchestration	Temporal or equivalent workflow engine	Supports durable retries, long-running workflows, and stateful execution.
Agent orchestration (optional)	LangGraph or similar; LangChain agents for higher-level LLM/tool loops [46,47]	Future dynamic branching, durable execution, and human-in-the-loop checkpoints. POC v1 uses deterministic Python orchestration.
Messaging/eventing	RabbitMQ or NATS	Decouples sensing ingestion from agent execution.
Structured storage	PostgreSQL with TimescaleDB for time-series	Stores longitudinal wearable and respiratory trajectories.
Artifact/object storage	S3 or MinIO	Stores reports, figures, audit artifacts, model metadata, and output packages.
Identity and access	OIDC, role-based access control, e.g., Keycloak	Required before handling real clinical data across user roles.
Secrets management	Vault or Cloud Secret Manager	Removes credentials from code and configuration files.
Observability	OpenTelemetry, Prometheus, and Grafana	Provides end-to-end traceability of agent decisions, latency, and failures.
Governance and audit	Append-only audit trail and policy engine	Supports Privacy and Safety Governance Agent responsibilities.
Clinical interoperability	FHIR/HL7 output adapters	Enables EHR-facing reporting and integration with clinical systems.
Deployment	Docker Compose for staging; Kubernetes for scalable environments	Separates research, staging, and controlled pilot environments.
CI/CD and assurance	GitHub Actions with tests, schema checks, and reproducibility checks	Adds regression testing for scoring, Safety Gate behavior, and schema compatibility.

**Table 8:** Summary of the 139-patient proof-of-concept validation cohort.

Result	Value	Interpretation
Validation cohort	139 patients	Patients with complete bilateral lung CT metrics
CT reliability range	0.0177–0.8600	Broad segmentation-quality spectrum
Mean CT reliability	0.4065	Moderate overall imaging reliability
Median CT reliability	0.4292	Consistent with heterogeneous CT quality
Management divergence from CT-only	78/139 (56.1%)	Multimodal POC changed review priority versus CT-only
Direct safety gate escalations	41/139 (29.5%)	Explicit uncertainty escalation requiring human review
High-priority or high-uncertainty assignments	111/139 (79.9%)	Conservative review behavior in monitoring context

**Table 9:** Review-priority distribution in the 139-patient proof-of-concept cohort.

Review-Priority Tier	N	%	Interpretation
High priority	41	29.5	Immediate escalation; multimodal signals concordantly elevated
High uncertainty	70	50.4	Manual review required because of uncertainty or modality conflict
Moderate priority	19	13.7	Watchful monitoring or scheduled clinician review
Low priority	9	6.5	Routine surveillance; signals concordant and stable

**Table 10:** CT quality stratification and Safety Gate behavior.

CT Quality Tier	N	%	Mean Reliability	Safety Gate Rate	Management-Change Rate
Excellent Dice ≥ 0.80	19	13.7	0.682	15.8%	42.1%
Good Dice 0.60–0.79	31	22.3	0.448	25.8%	51.6%
Moderate Dice 0.40–0.59	51	36.7	0.295	31.4%	58.8%
Poor Dice 0.20–0.39	28	20.1	0.127	39.3%	67.9%
Very poor Dice < 0.20	10	7.2	0.029	60.0%	80.0%

**Table 11:** Baseline and adversarial conflict-testing summary.

Evaluation Component	Result	Interpretation
Deterministic aligned overlays	Simple baselines can perform strongly when all signals agree	Confirms expected behavior but does not prove clinical superiority
Imaging-only baseline	Fails to represent deterioration invisible to CT	Shows why CT alone is insufficient for longitudinal monitoring
Non-agentic late fusion	Can average multimodal risk but lacks explicit reliability logic	Useful comparator but less auditable than bounded-agent fusion
Adversarial conflict cases	11/15 cases showed multi-method disagreement	Demonstrates that modality conflict changes review-priority behavior
OncoSense-Agent POC	Integrates reliability, Safety Gate logic, and explanations	Provides auditable review-priority generation under uncertainty

**Table 12:** Summary of the three illustrative cases.

Case	CT-Only Interpretation	OncoSense-Agent Interpretation	Main Architecture Value
RC-047	Moderate	High	Detects respiratory and wearable deterioration despite non-alarming CT
RC-102	Low	Moderate with data-quality flag	Identifies wearable uncertainty and requests verification
RC-203	Moderate	High	Detects comorbidity and treatment-toxicity interaction despite acceptable imaging

**Table 13:** Cross-case architecture value.

Architecture Behavior	RC-047	RC-102	RC-203
Detects deterioration not visible in CT	Yes	Partial	Yes
Handles signal reliability	Yes	Strongly yes	Yes
Adds treatment-context reasoning	Yes	Yes	Strongly yes
Produces a different output than CT-only	Yes	Yes	Yes
Main value	Escalation	Verification	Early safety intervention

## Data Availability

The private clinical CT dataset and patient-level imaging data cannot be publicly released because of patient confidentiality, consent limitations, and institutional data governance restrictions. Aggregated segmentation metrics, proof-of-concept logic, deterministic overlay definitions, and non-identifiable summary results can be made available upon reasonable request, subject to institutional approval.

## Abbreviations

The following abbreviations are used in this manuscript:

**Abbreviation**	**Meaning**
AI	Artificial intelligence
CDSS	Clinical decision support system
COPD	Chronic obstructive pulmonary disease
CT	Computed tomography
DICOM	Digital Imaging and Communications in Medicine
EHR	Electronic health record
FEV1	Forced expiratory volume in one second
FHIR	Fast Healthcare Interoperability Resources
HD95	95th percentile Hausdorff distance
HR	Heart rate
HRV	Heart-rate variability
OAR	Organ at risk
PEF	Peak expiratory flow
PRO	Patient-reported outcome
RPM	Remote patient monitoring
RTSTRUCT	Radiotherapy structure set
SpO2	Peripheral oxygen saturation
XAI	Explainable artificial intelligence
6MWT	Six-minute walk test
CAT	COPD Assessment Test
mMRC	Modified Medical Research Council dyspnoea scale
